# Supplementary material for: Identification of miRNA signatures for kidney renal clear cell carcinoma using the tensor-decomposition method
Source: Sci Rep. 2020 Sep 16;10:15149. doi: 10.1038/s41598-020-71997-6 (PMC7494921; doi:10.1038/s41598-020-71997-6)
Supplement: Supplementary file 2 — Supplementary Information 2. [file 41598_2020_71997_MOESM2_ESM.zip › Title_page_for_supplementary_materials.docx]

Identification of miRNA signatures for kidney renal clear cell carcinoma using the tensor-decomposition method

Ka-Lok Ng ^1, 2^ and Y-h Taguchi ^3,^*

*^1^ Department of Bioinformatics and Medical Engineering Asia University, Taichung, Taiwan*

*^2^ Department of Medical Research, China Medical University Hospital, China Medical University, Taiwan*

*^3^ Department of Physics Chuo University, 1-13-27 Kasuga Bunky-ku, Tokyo 112-8551, Japan;*

^1^ [ppiddi@gmail.com](mailto:ppiddi@gmail.com) ^3,^* [tag@granular.com](mailto:tag@granular.com)

* corresponding author
