# Supplementary figures and images for: Identification of miRNA signatures for kidney renal clear cell carcinoma using the tensor-decomposition method
Source: Sci Rep. 2020 Sep 16;10:15149. doi: 10.1038/s41598-020-71997-6 (PMC7494921; doi:10.1038/s41598-020-71997-6)

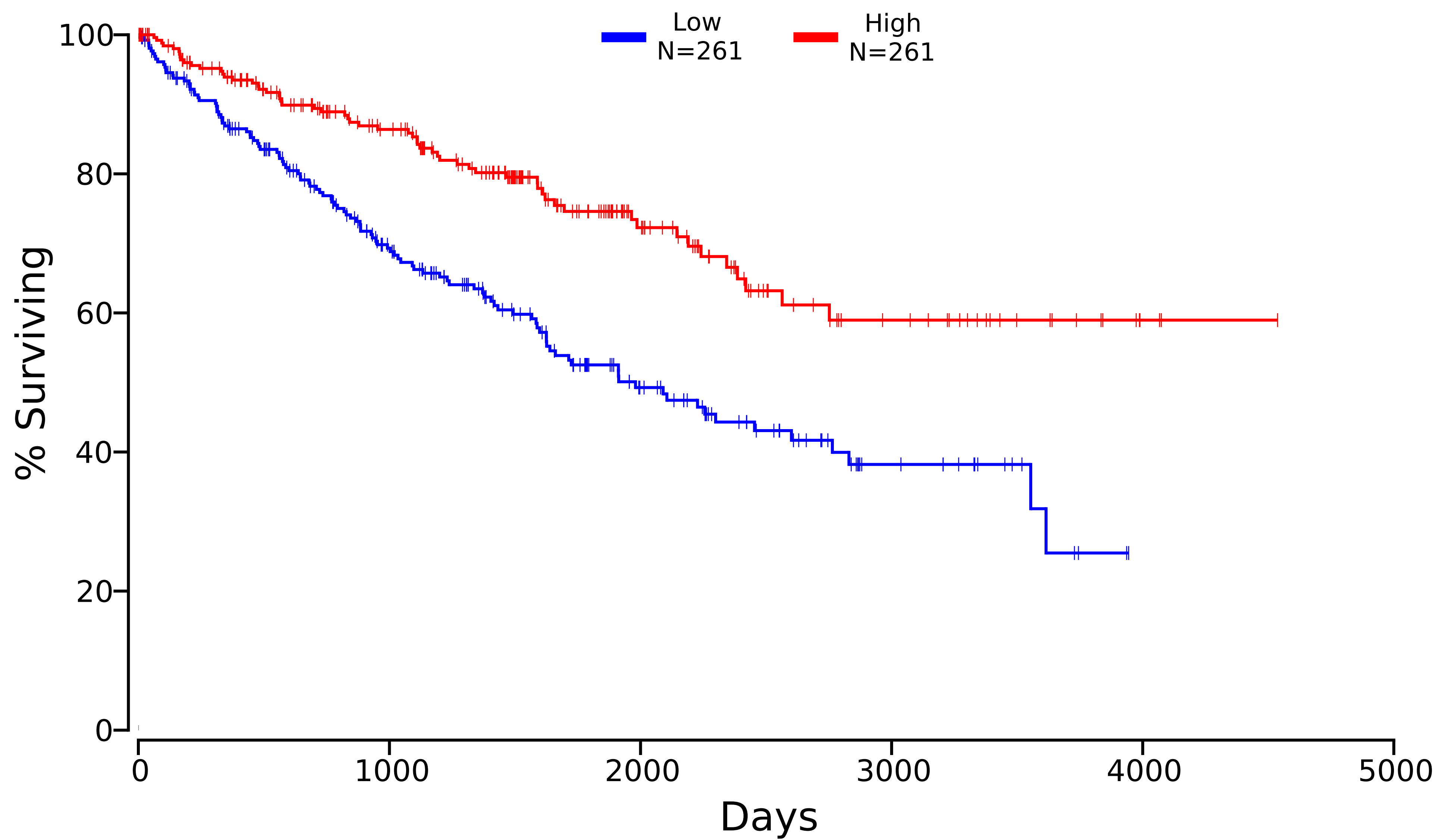

Supplement: Supplementary file 2 — Supplementary Information 2. [file 41598_2020_71997_MOESM2_ESM.zip › Suppl figure KM plot/PODXL_KIRC_5420_50_50.pdf]

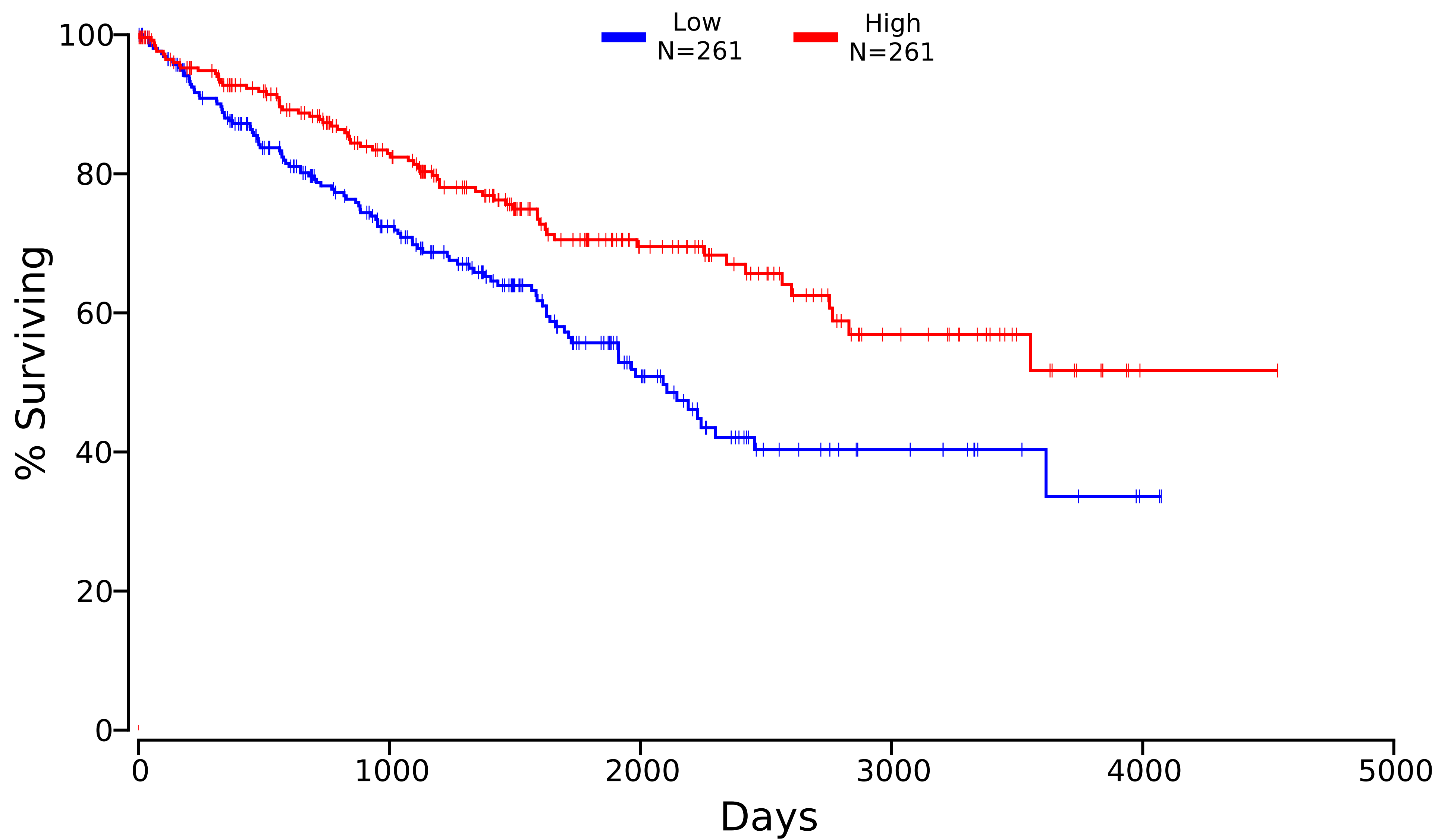

Supplement: Supplementary file 2 — Supplementary Information 2. [file 41598_2020_71997_MOESM2_ESM.zip › Suppl figure KM plot/GATM_KIRC_2628_50_50.pdf]

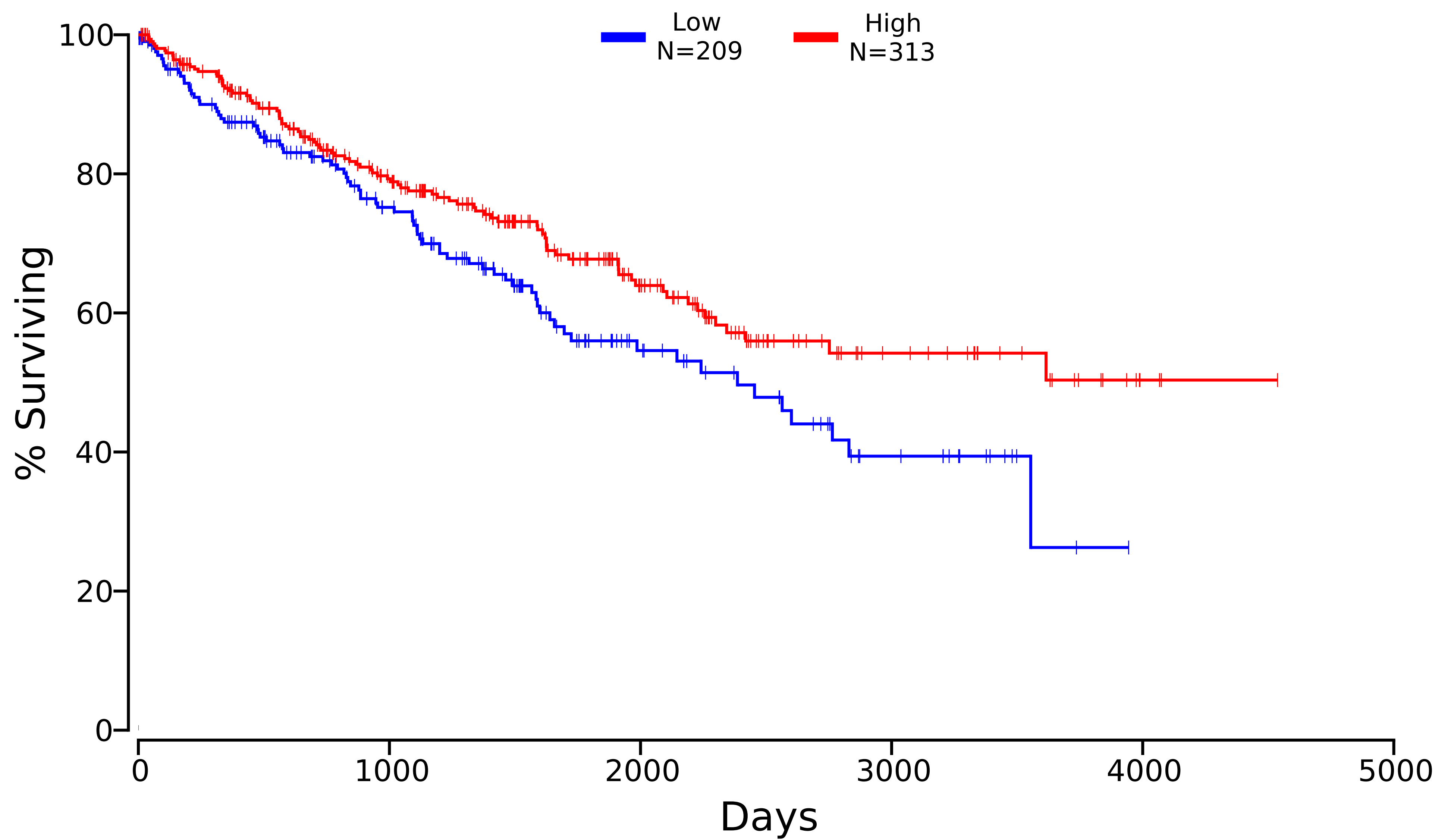

Supplement: Supplementary file 2 — Supplementary Information 2. [file 41598_2020_71997_MOESM2_ESM.zip › Suppl figure KM plot/ATP1A1_KIRC_476_40_60.pdf]

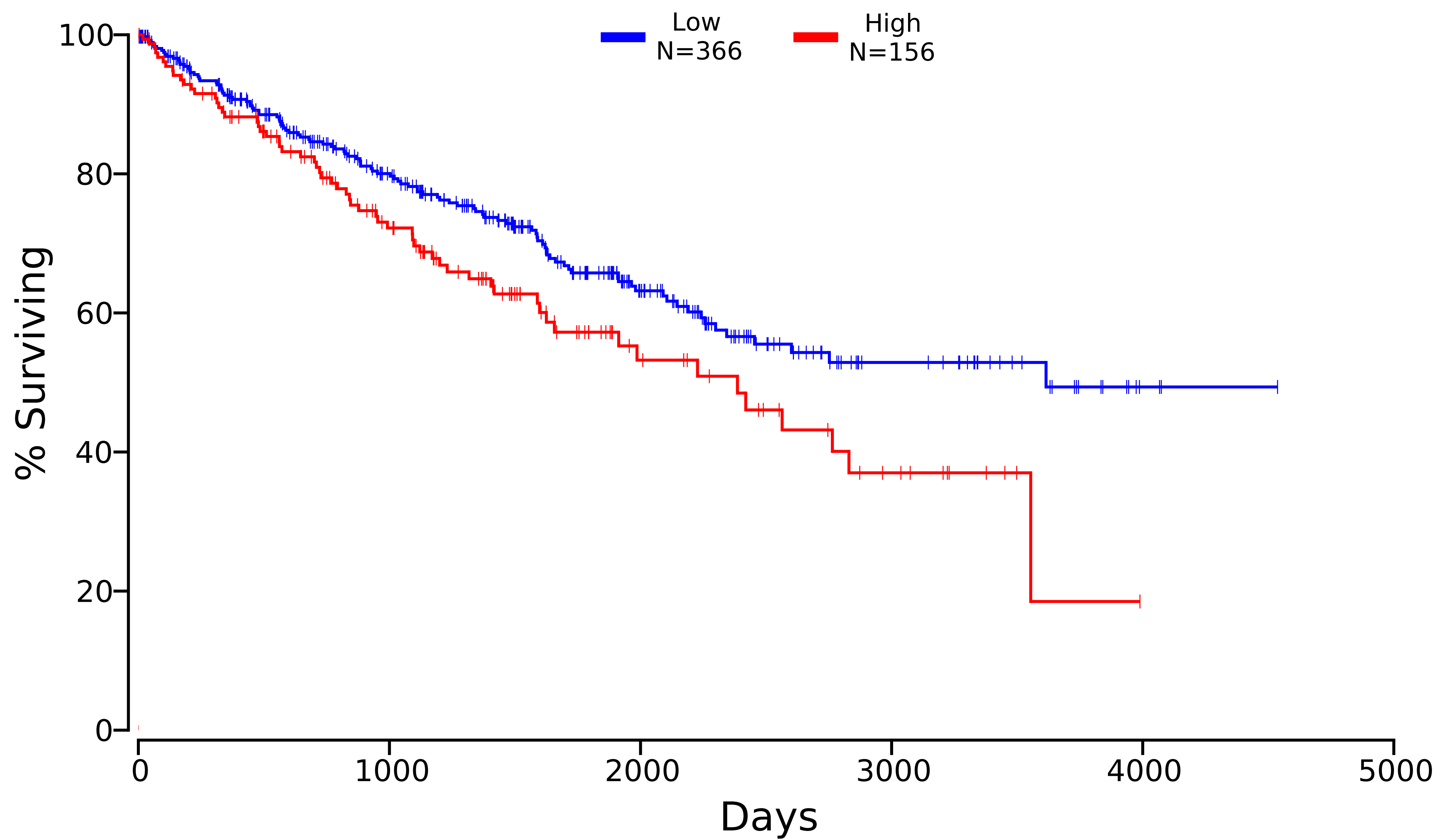

Supplement: Supplementary file 2 — Supplementary Information 2. [file 41598_2020_71997_MOESM2_ESM.zip › Suppl figure KM plot/VEGFA_KIRC_7422_70_30.pdf]

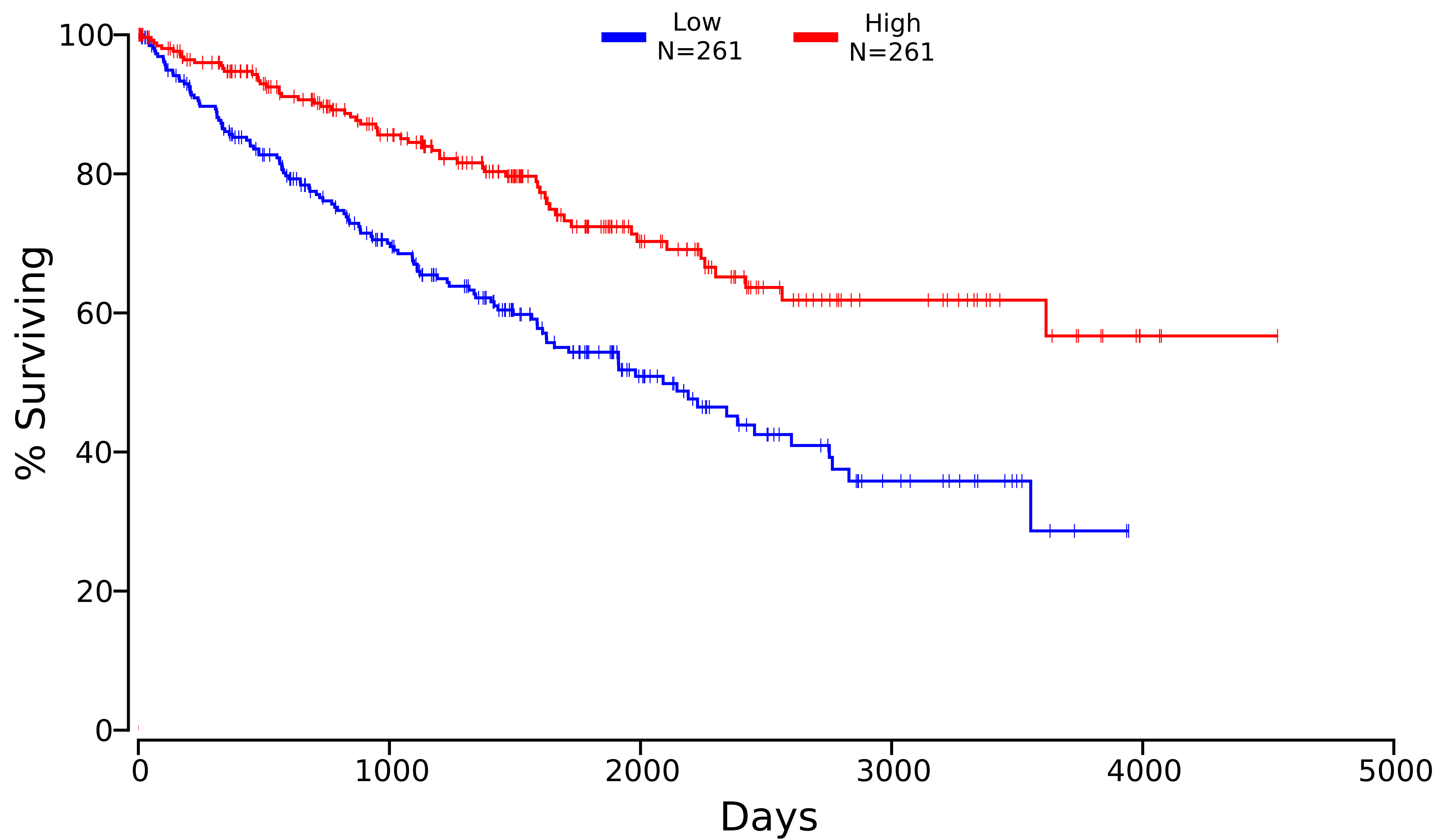

Supplement: Supplementary file 2 — Supplementary Information 2. [file 41598_2020_71997_MOESM2_ESM.zip › Suppl figure KM plot/APP_KIRC_351_50_50.pdf]

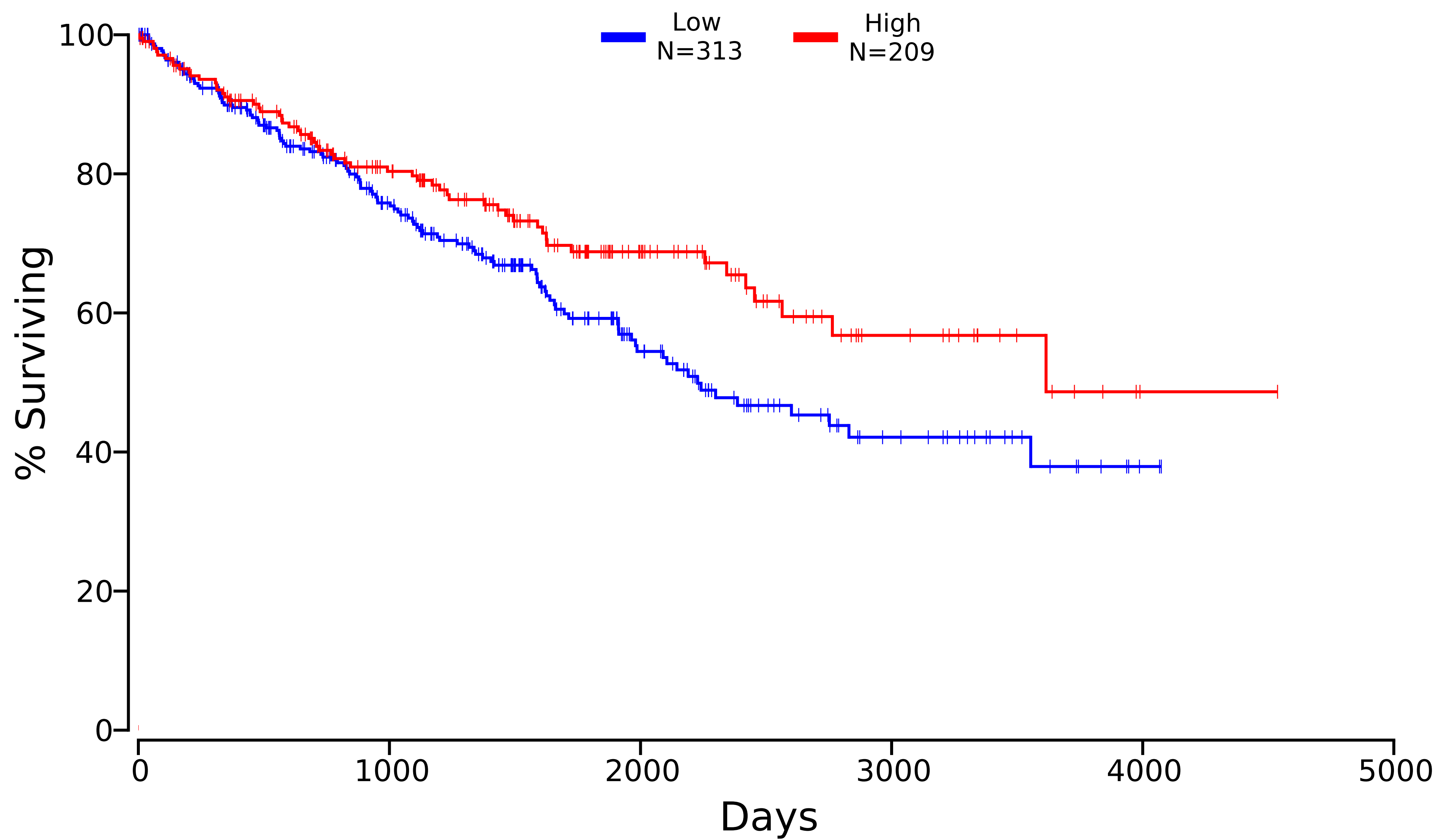

Supplement: Supplementary file 2 — Supplementary Information 2. [file 41598_2020_71997_MOESM2_ESM.zip › Suppl figure KM plot/NDRG1_KIRC_10397_60_40.pdf]

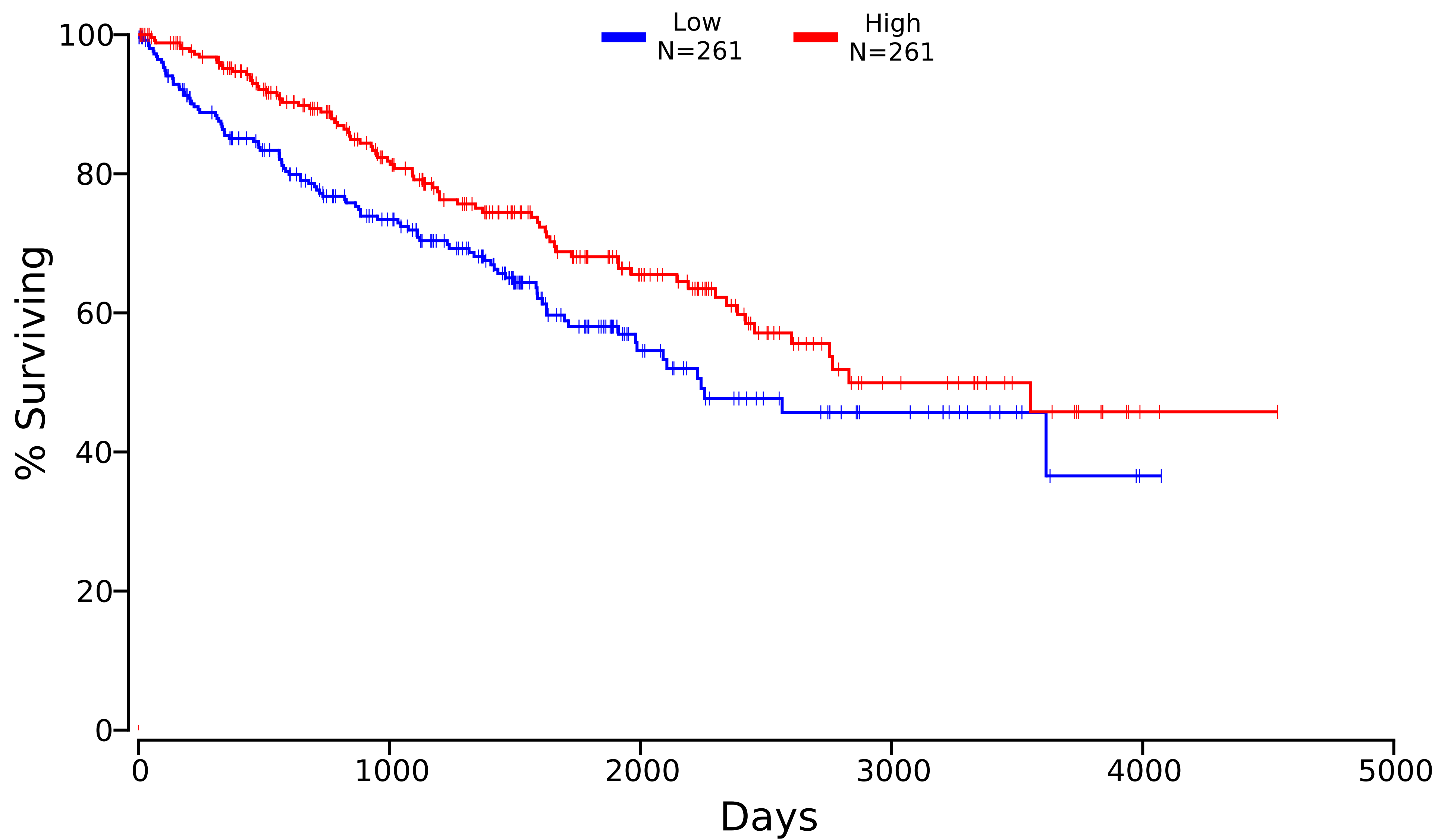

Supplement: Supplementary file 2 — Supplementary Information 2. [file 41598_2020_71997_MOESM2_ESM.zip › Suppl figure KM plot/ITM2B_KIRC_9445_50_50.pdf]

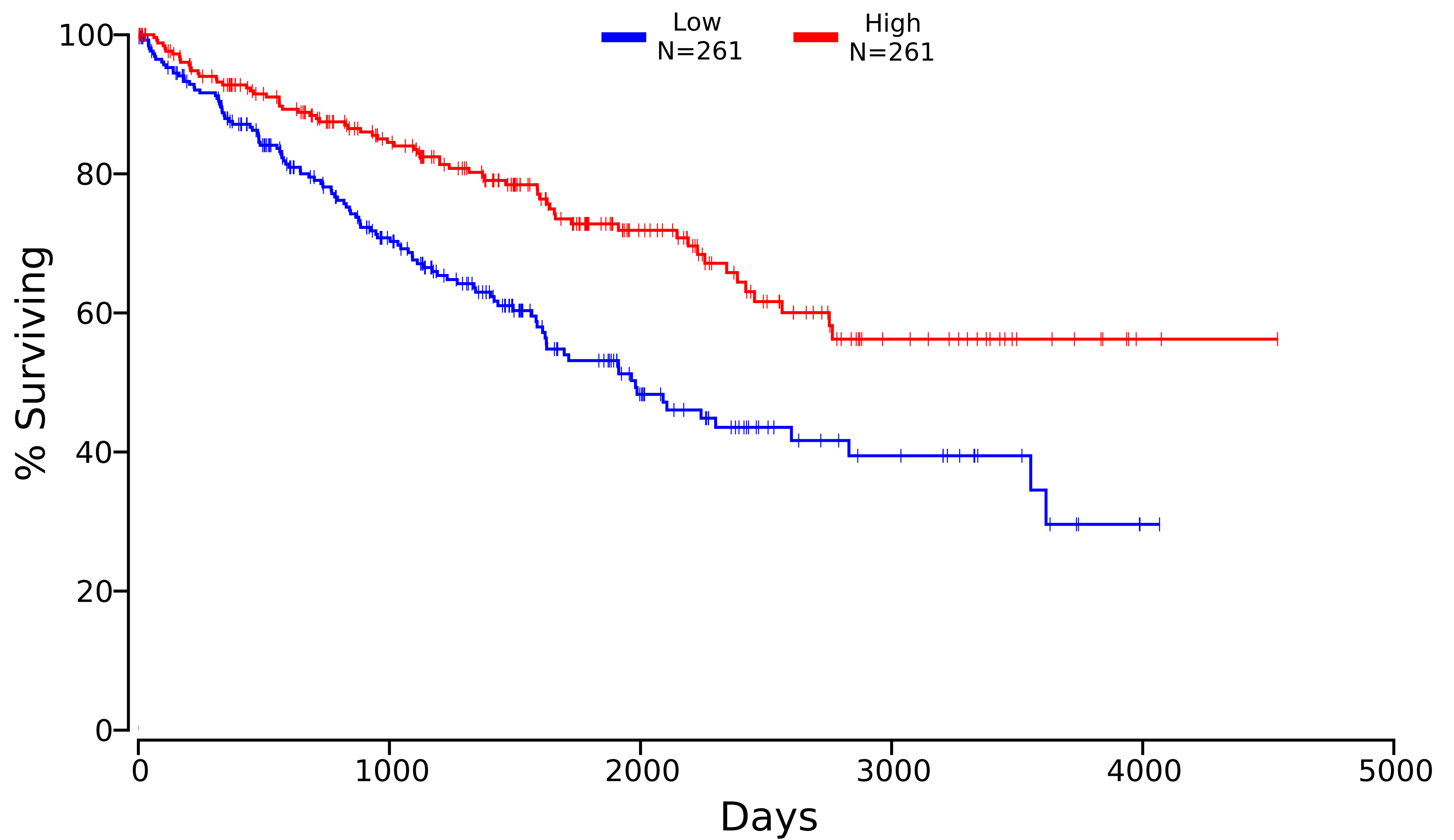

Supplement: Supplementary file 2 — Supplementary Information 2. [file 41598_2020_71997_MOESM2_ESM.zip › Suppl figure KM plot/PLIN2_KIRC_123_50_50.pdf]

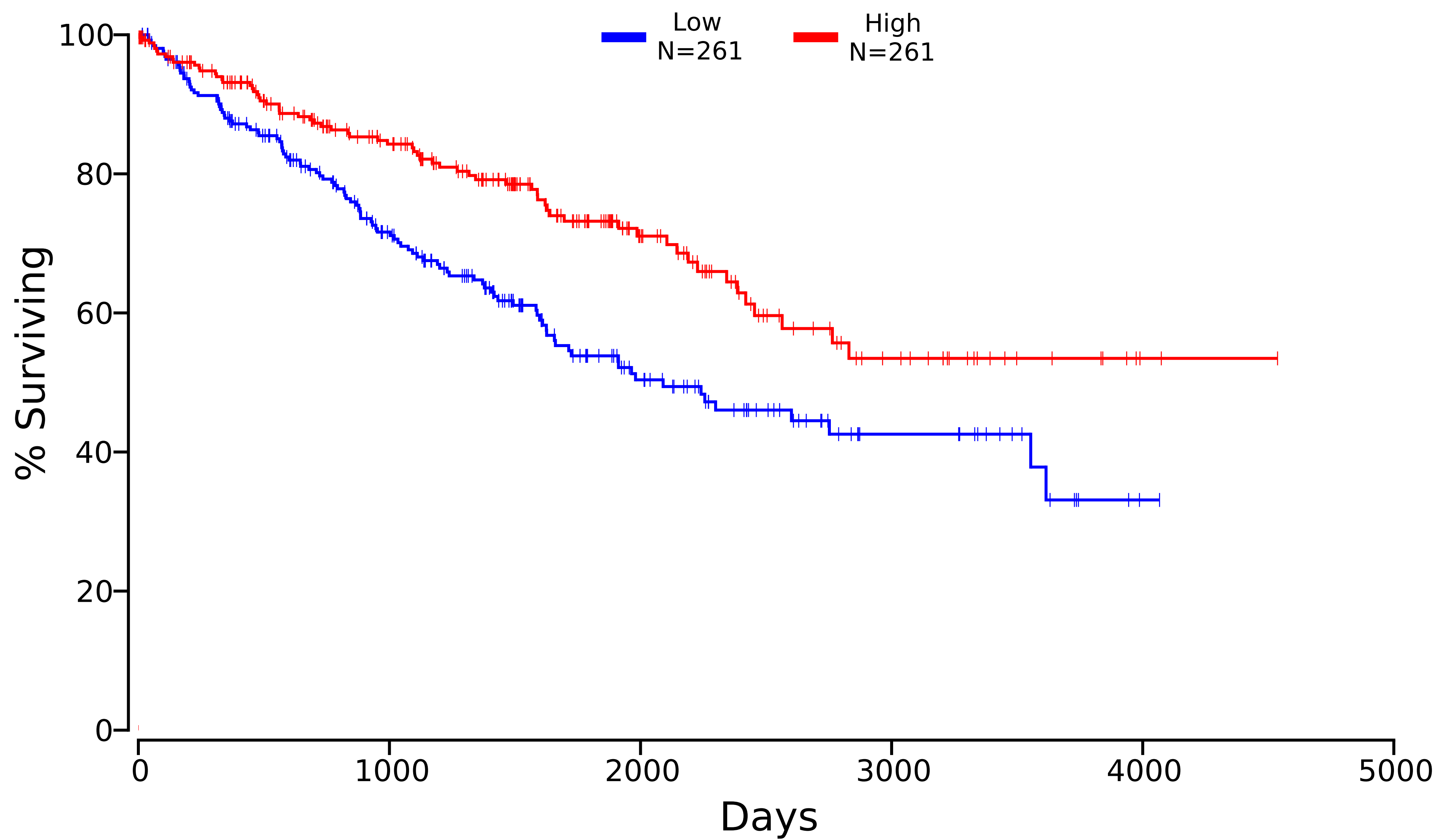

Supplement: Supplementary file 2 — Supplementary Information 2. [file 41598_2020_71997_MOESM2_ESM.zip › Suppl figure KM plot/CCND1_KIRC_595_50_50.pdf]

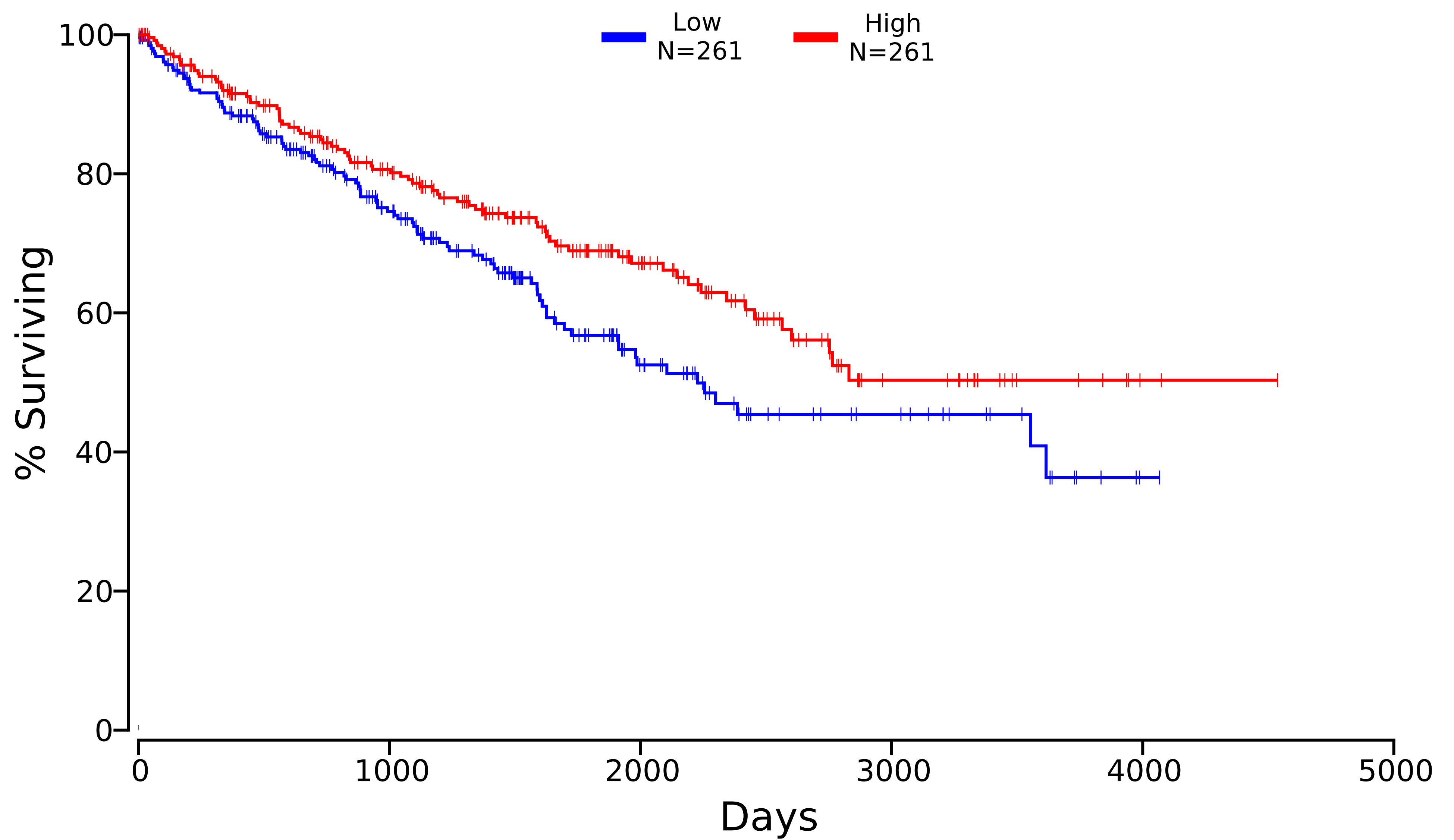

Supplement: Supplementary file 2 — Supplementary Information 2. [file 41598_2020_71997_MOESM2_ESM.zip › Suppl figure KM plot/ATP5A1_KIRC_498_50_50.pdf]

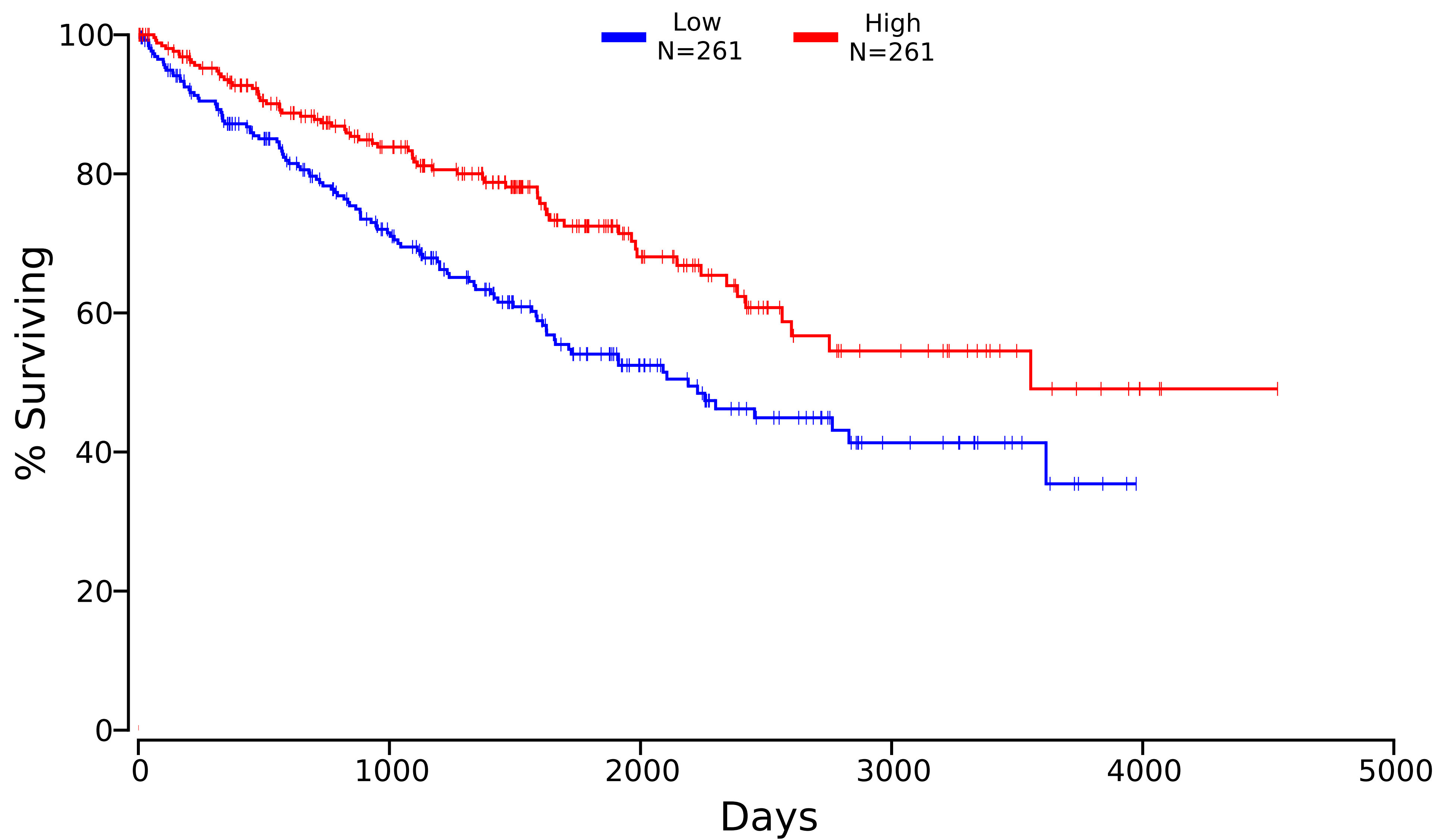

Supplement: Supplementary file 2 — Supplementary Information 2. [file 41598_2020_71997_MOESM2_ESM.zip › Suppl figure KM plot/PLVAP_KIRC_83483_50_50.pdf]

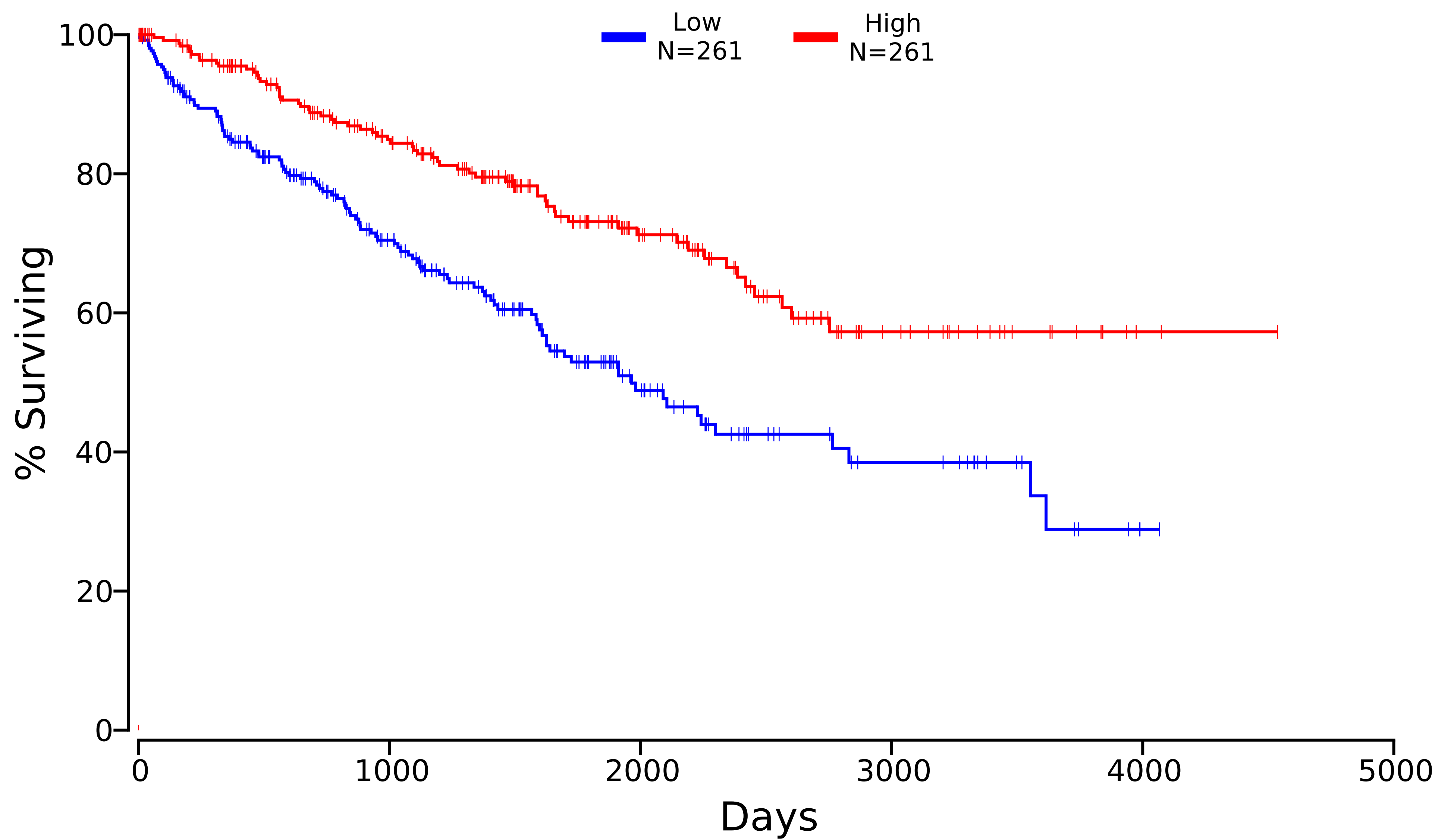

Supplement: Supplementary file 2 — Supplementary Information 2. [file 41598_2020_71997_MOESM2_ESM.zip › Suppl figure KM plot/ALDOB_KIRC_229_50_50.pdf]

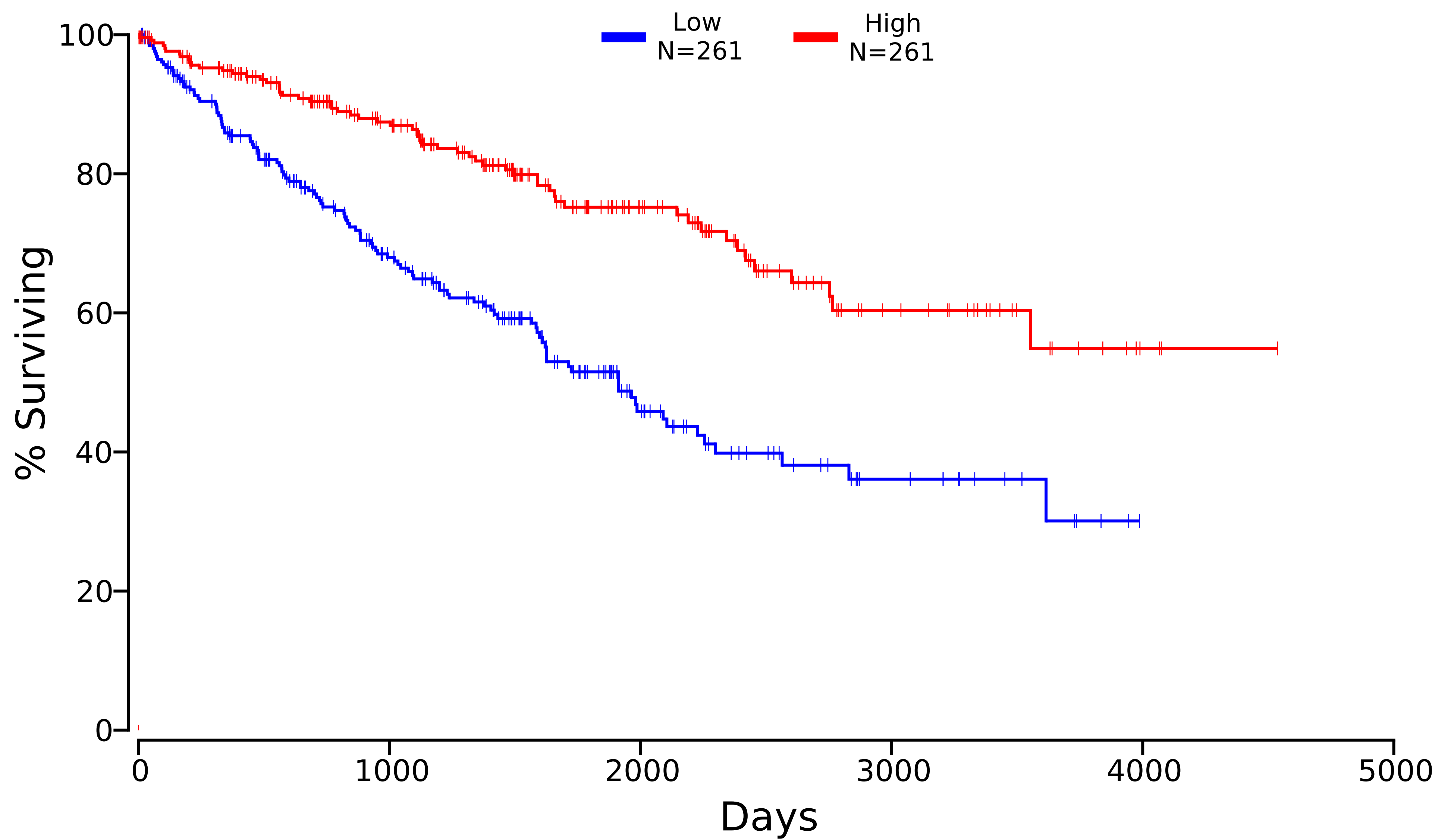

Supplement: Supplementary file 2 — Supplementary Information 2. [file 41598_2020_71997_MOESM2_ESM.zip › Suppl figure KM plot/AQP1_KIRC_358_50_50.pdf]

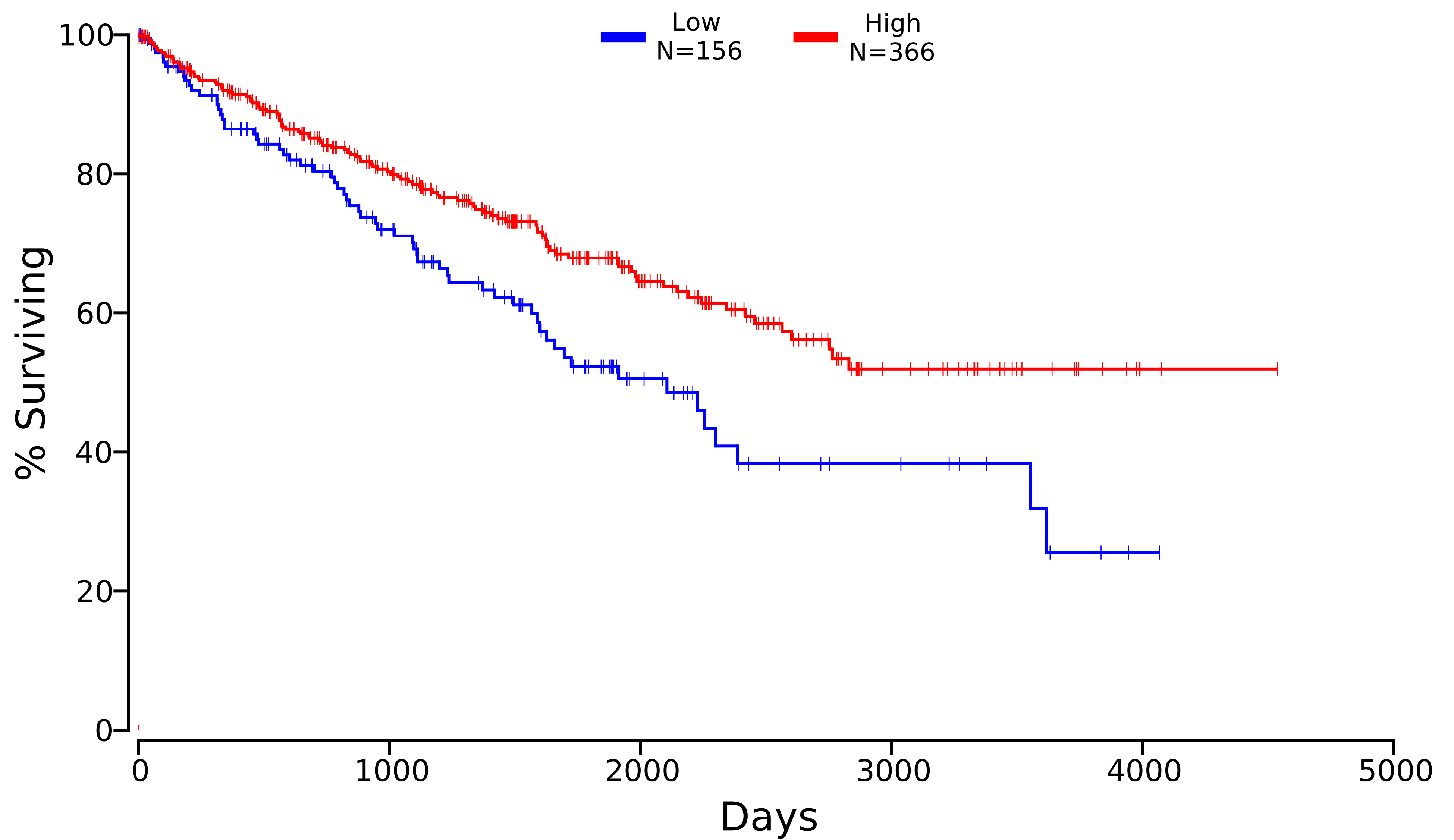

Supplement: Supplementary file 2 — Supplementary Information 2. [file 41598_2020_71997_MOESM2_ESM.zip › Suppl figure KM plot/ATP5B_KIRC_506_30_70.pdf]

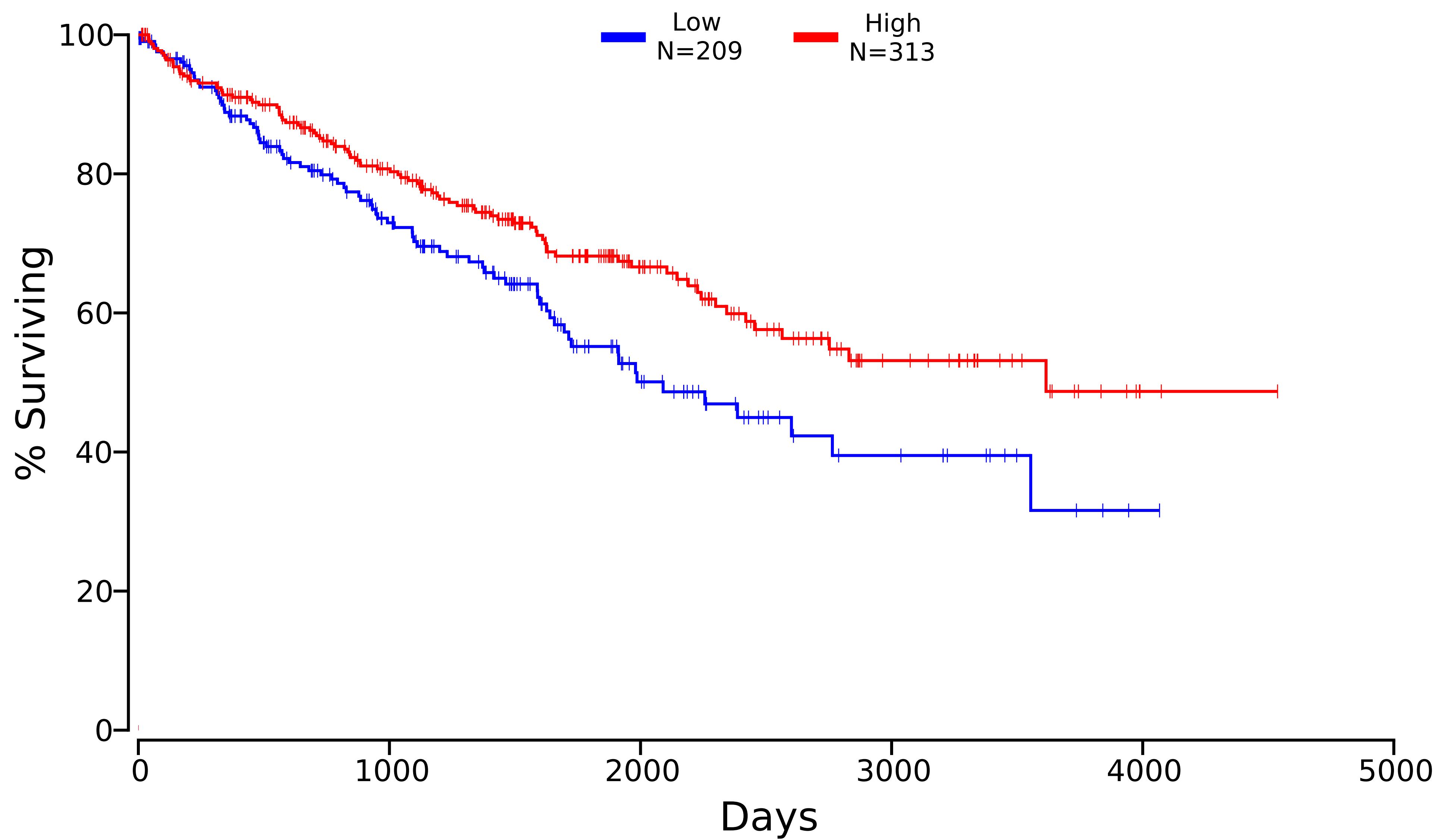

Supplement: Supplementary file 2 — Supplementary Information 2. [file 41598_2020_71997_MOESM2_ESM.zip › Suppl figure KM plot/HSPA8_KIRC_3312_40_60.pdf]

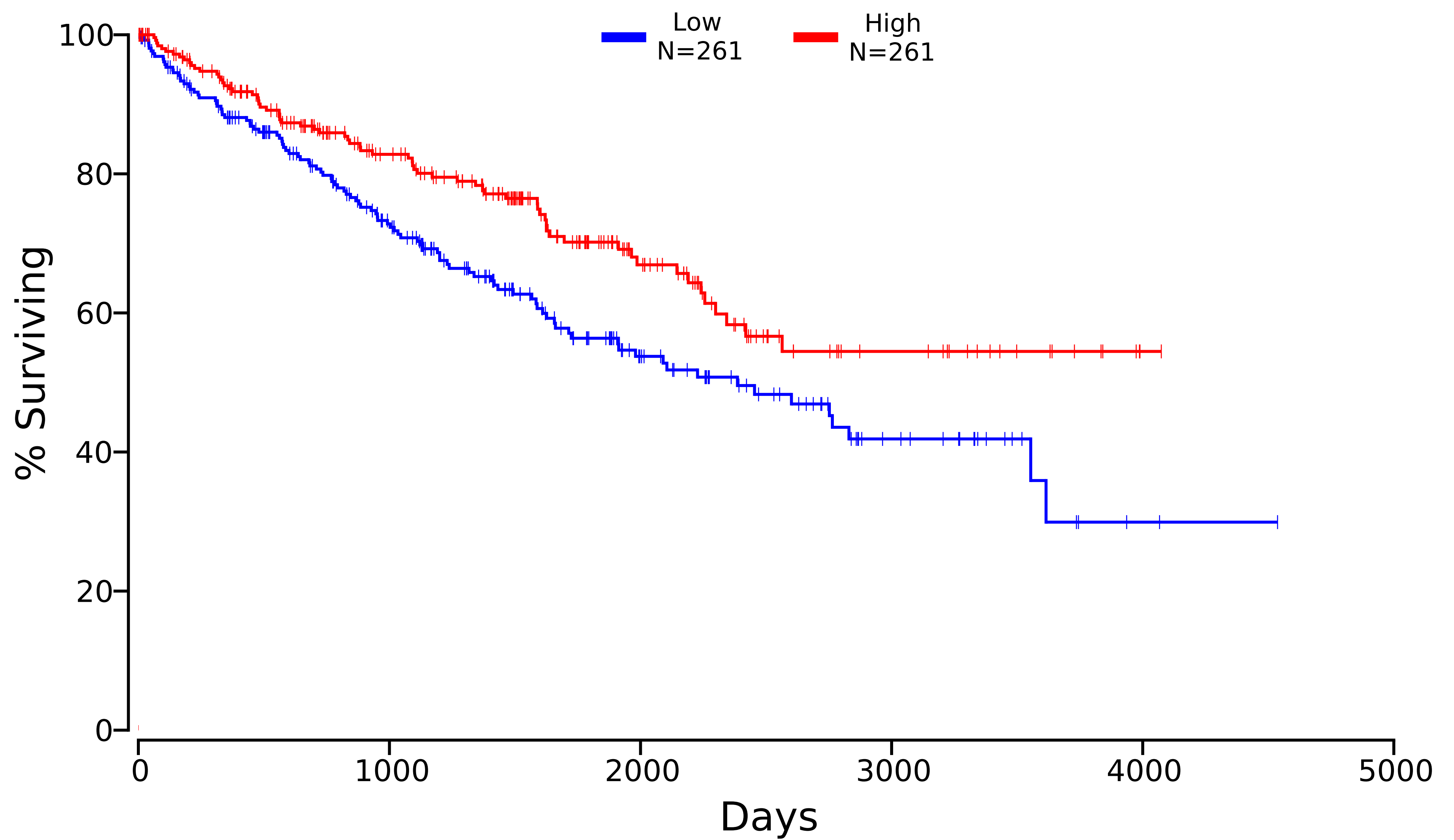

Supplement: Supplementary file 2 — Supplementary Information 2. [file 41598_2020_71997_MOESM2_ESM.zip › Suppl figure KM plot/VWF_KIRC_7450_50_50.pdf]

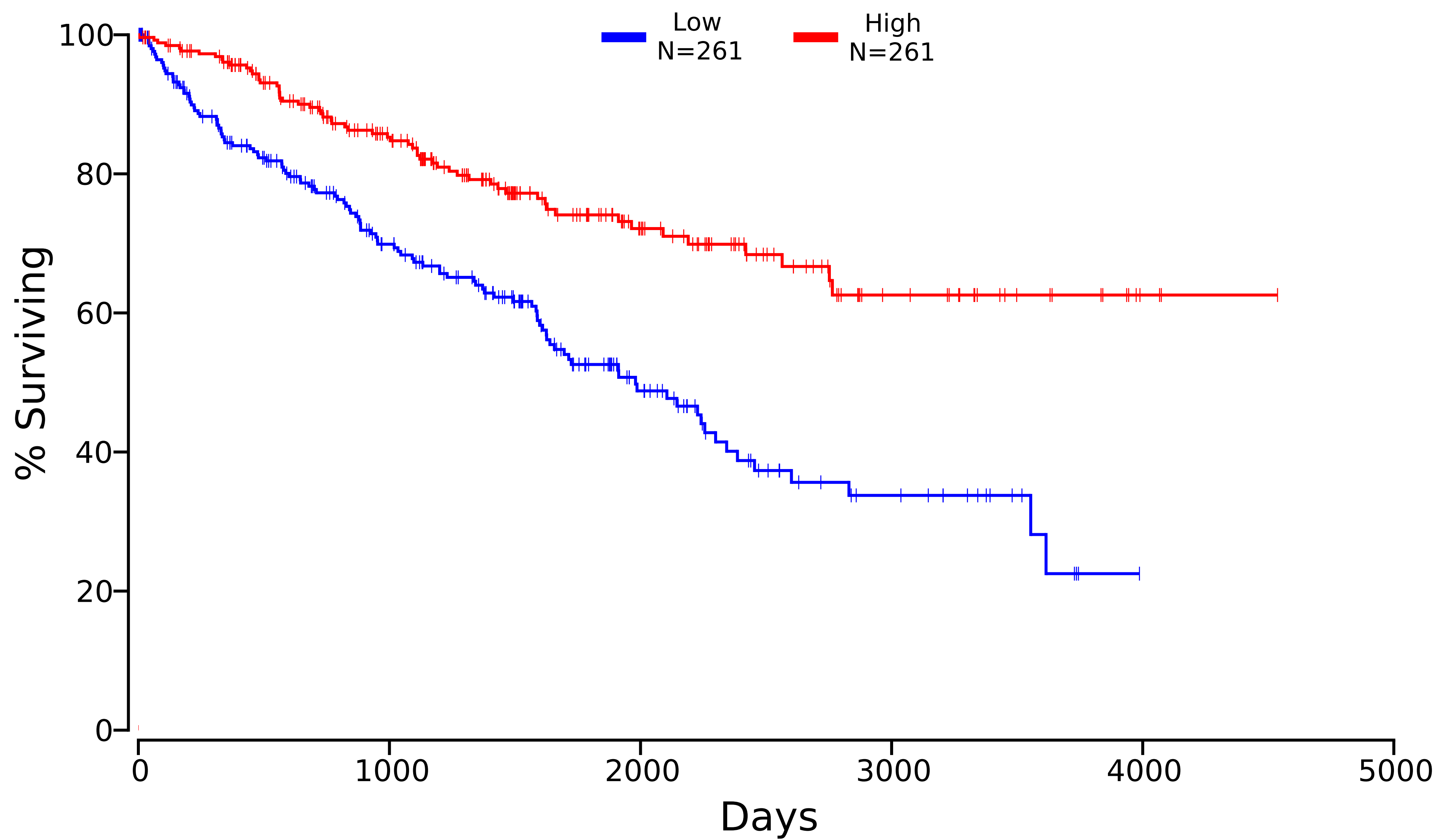

Supplement: Supplementary file 2 — Supplementary Information 2. [file 41598_2020_71997_MOESM2_ESM.zip › Suppl figure KM plot/CYFIP2_KIRC_26999_50_50.pdf]

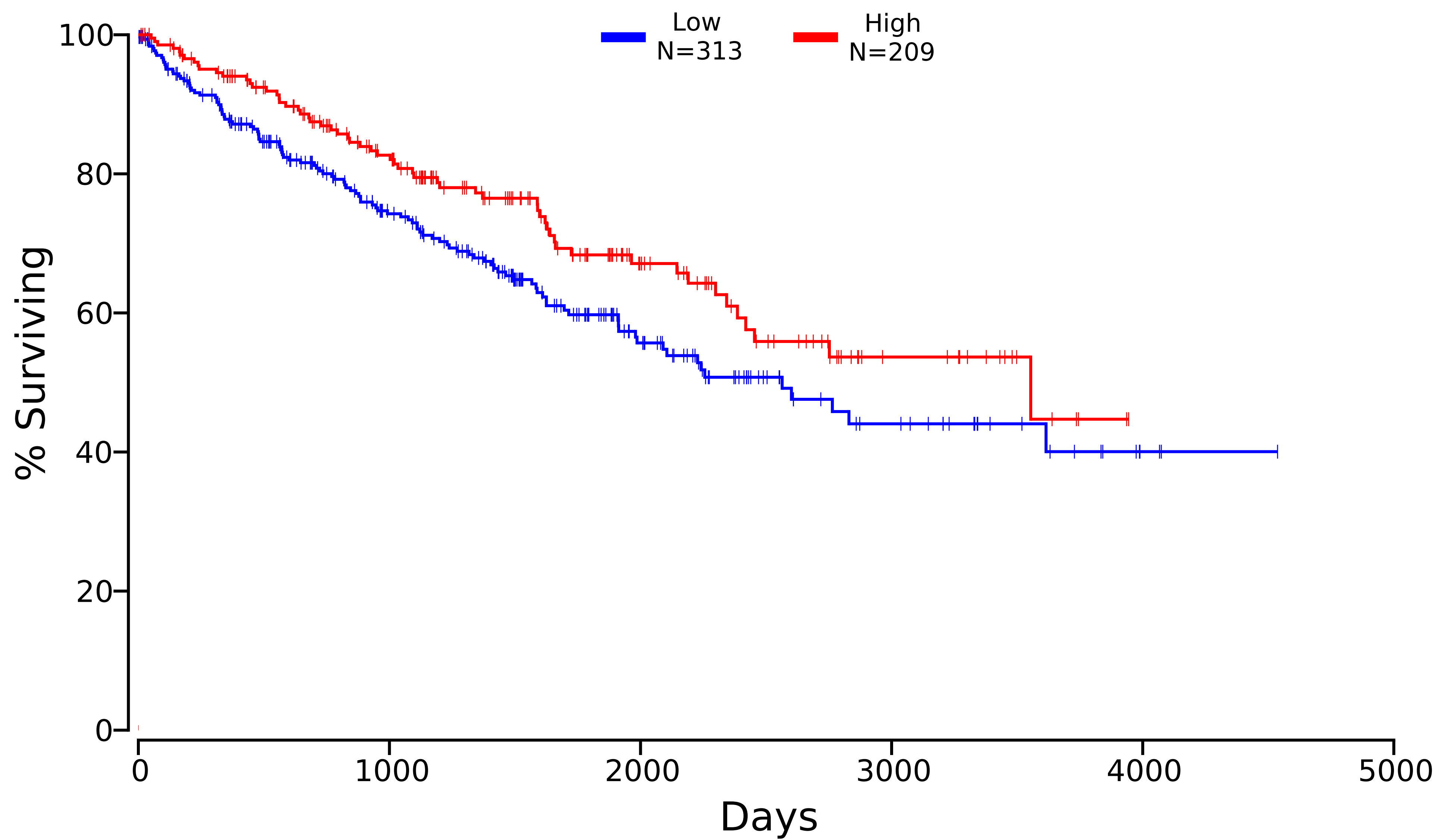

Supplement: Supplementary file 2 — Supplementary Information 2. [file 41598_2020_71997_MOESM2_ESM.zip › Suppl figure KM plot/AIF1L_KIRC_83543_60_40.pdf]

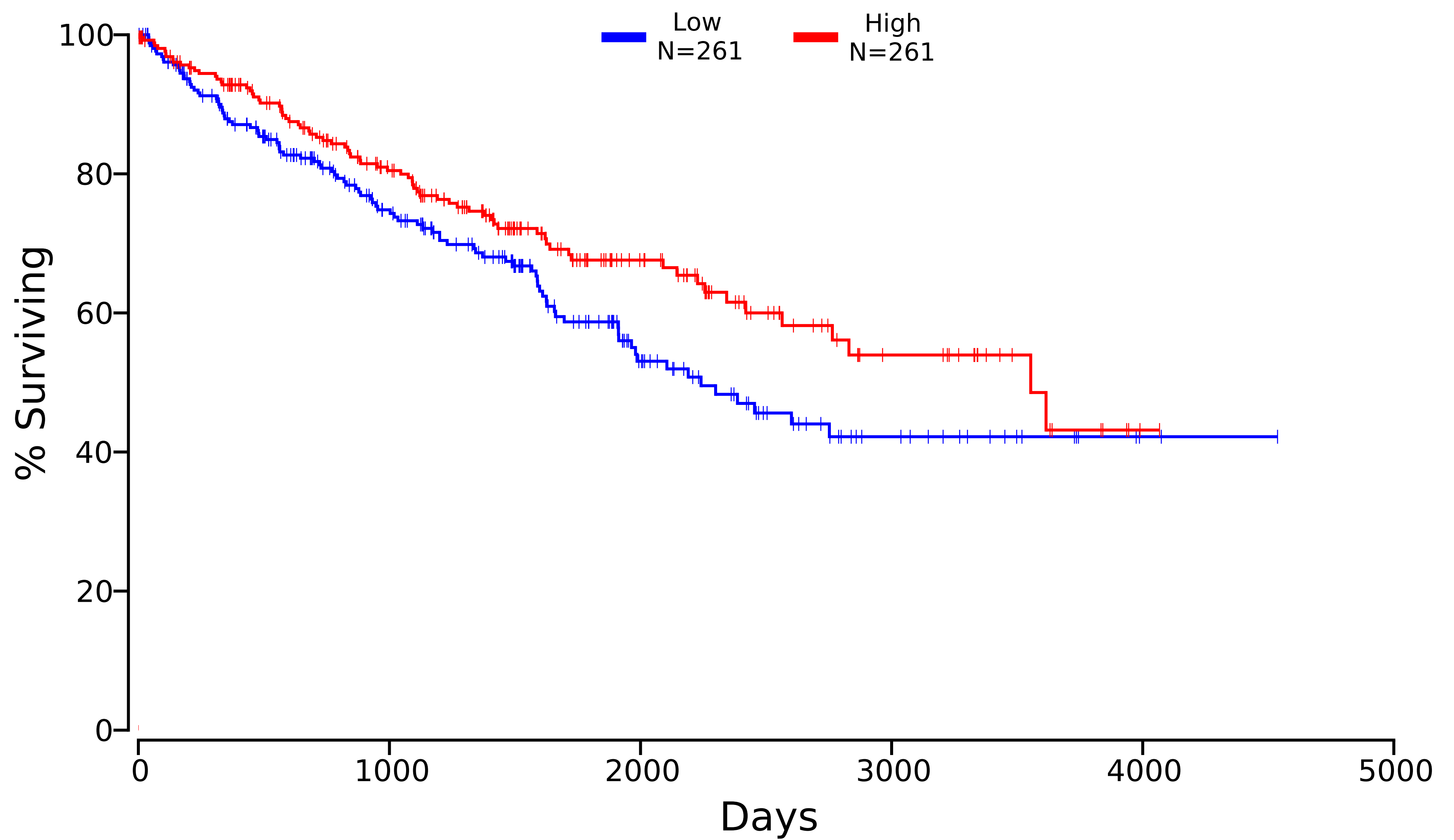

Supplement: Supplementary file 2 — Supplementary Information 2. [file 41598_2020_71997_MOESM2_ESM.zip › Suppl figure KM plot/ATP1B1_KIRC_481_50_50.pdf]

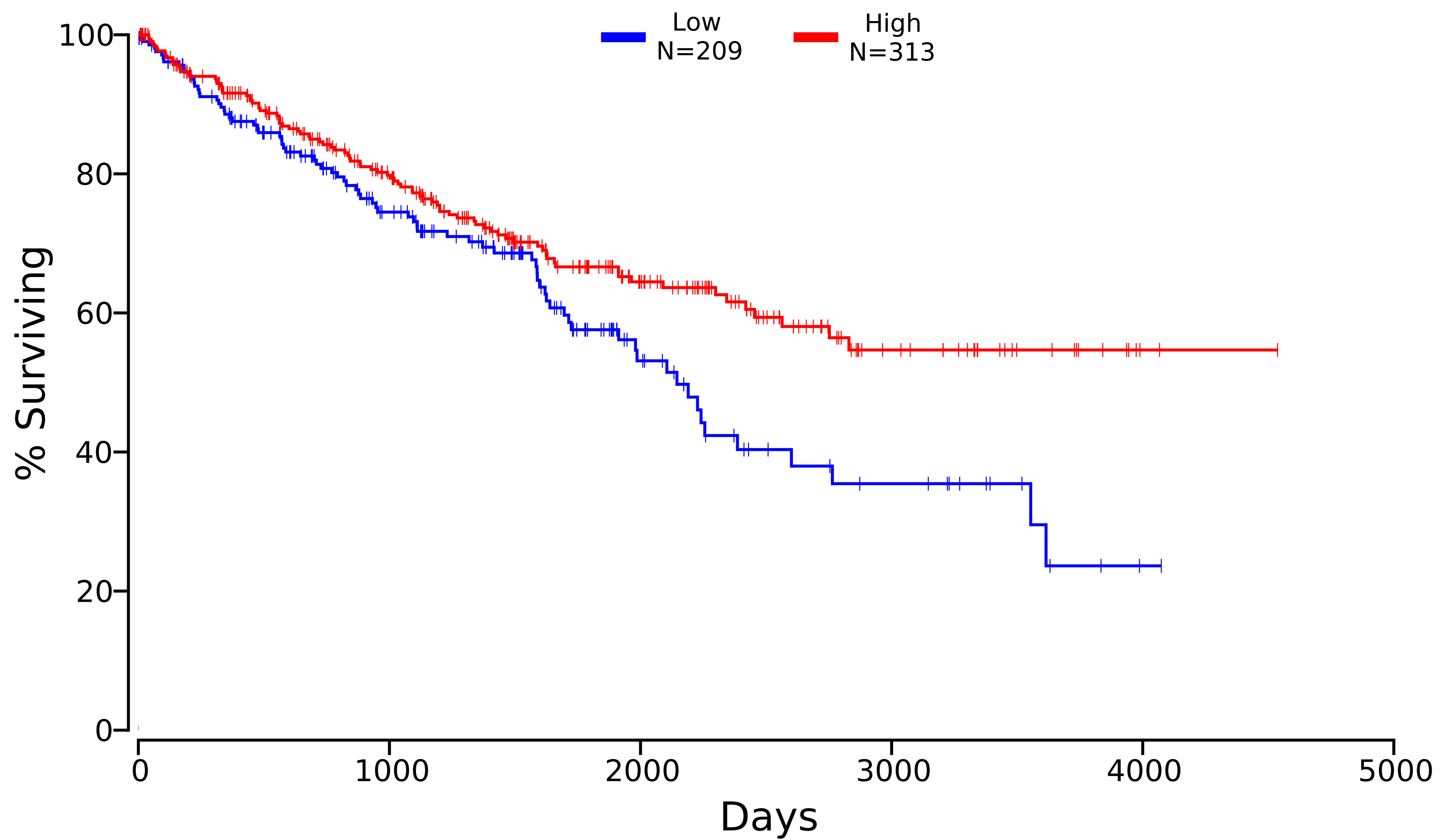

Supplement: Supplementary file 2 — Supplementary Information 2. [file 41598_2020_71997_MOESM2_ESM.zip › Suppl figure KM plot/TMBIM6_KIRC_7009_40_60.pdf]

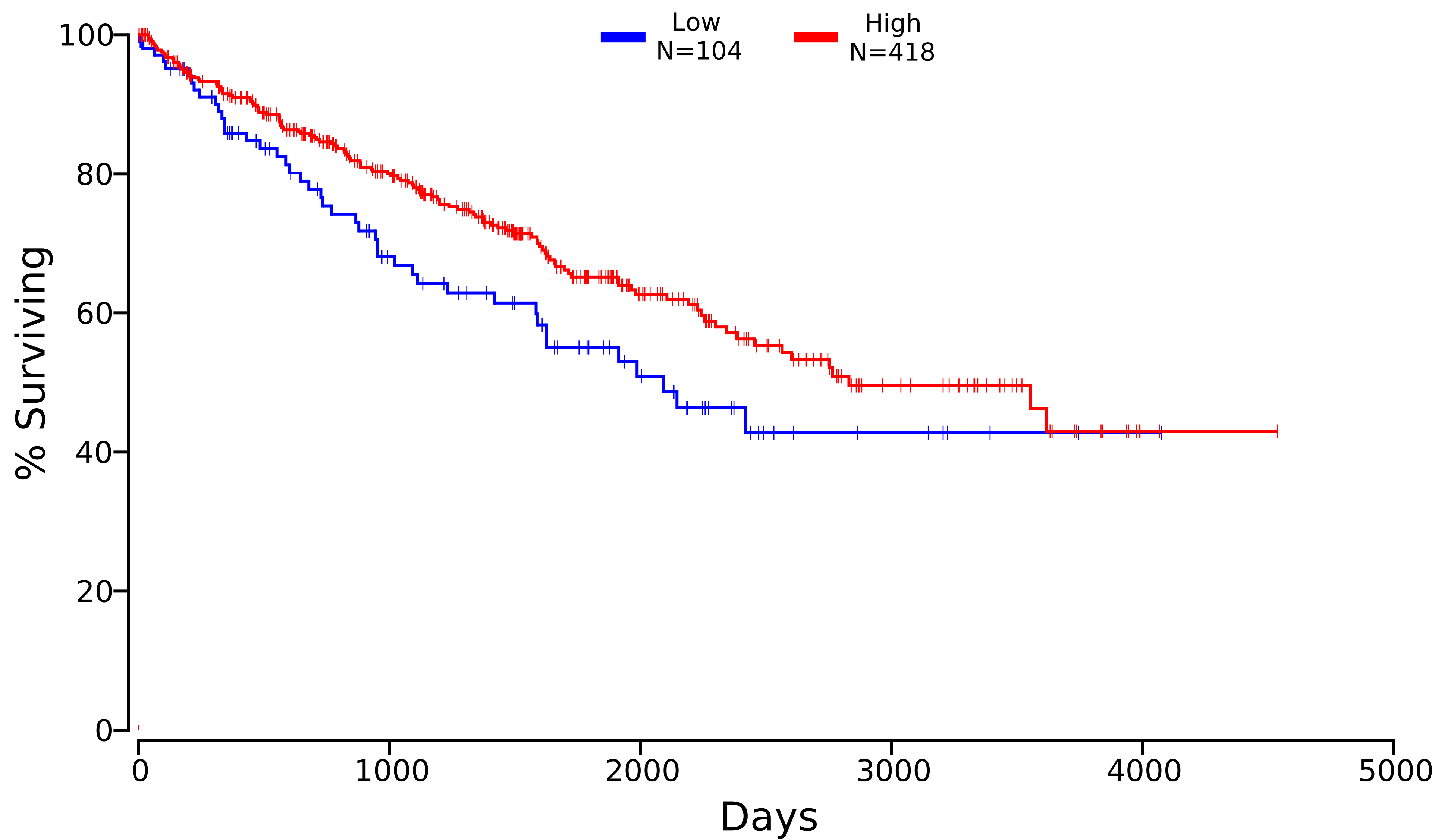

Supplement: Supplementary file 2 — Supplementary Information 2. [file 41598_2020_71997_MOESM2_ESM.zip › Suppl figure KM plot/HLA-DRA_KIRC_3122_20_80.pdf]

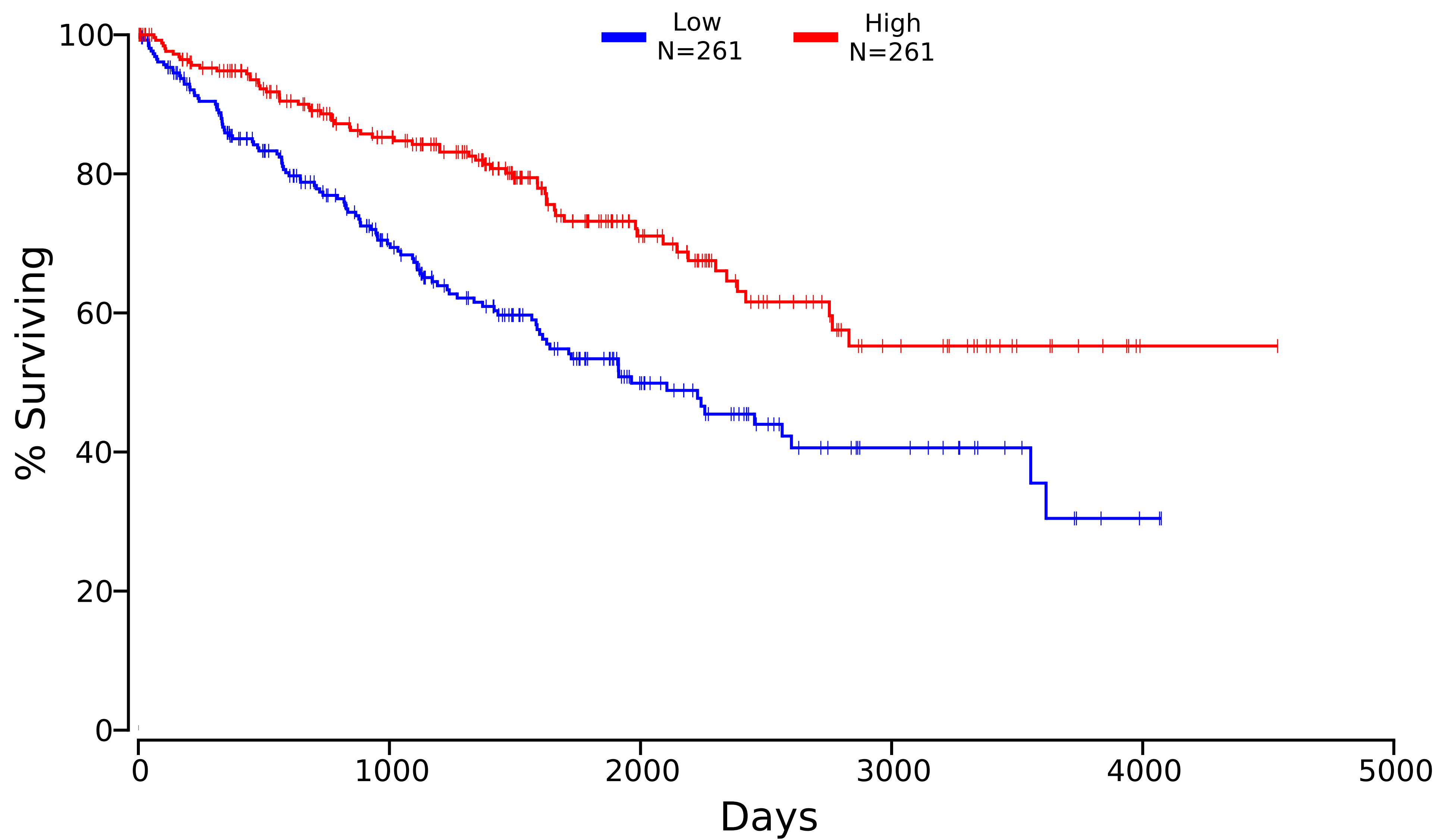

Supplement: Supplementary file 2 — Supplementary Information 2. [file 41598_2020_71997_MOESM2_ESM.zip › Suppl figure KM plot/PCK1_KIRC_5105_50_50.pdf]

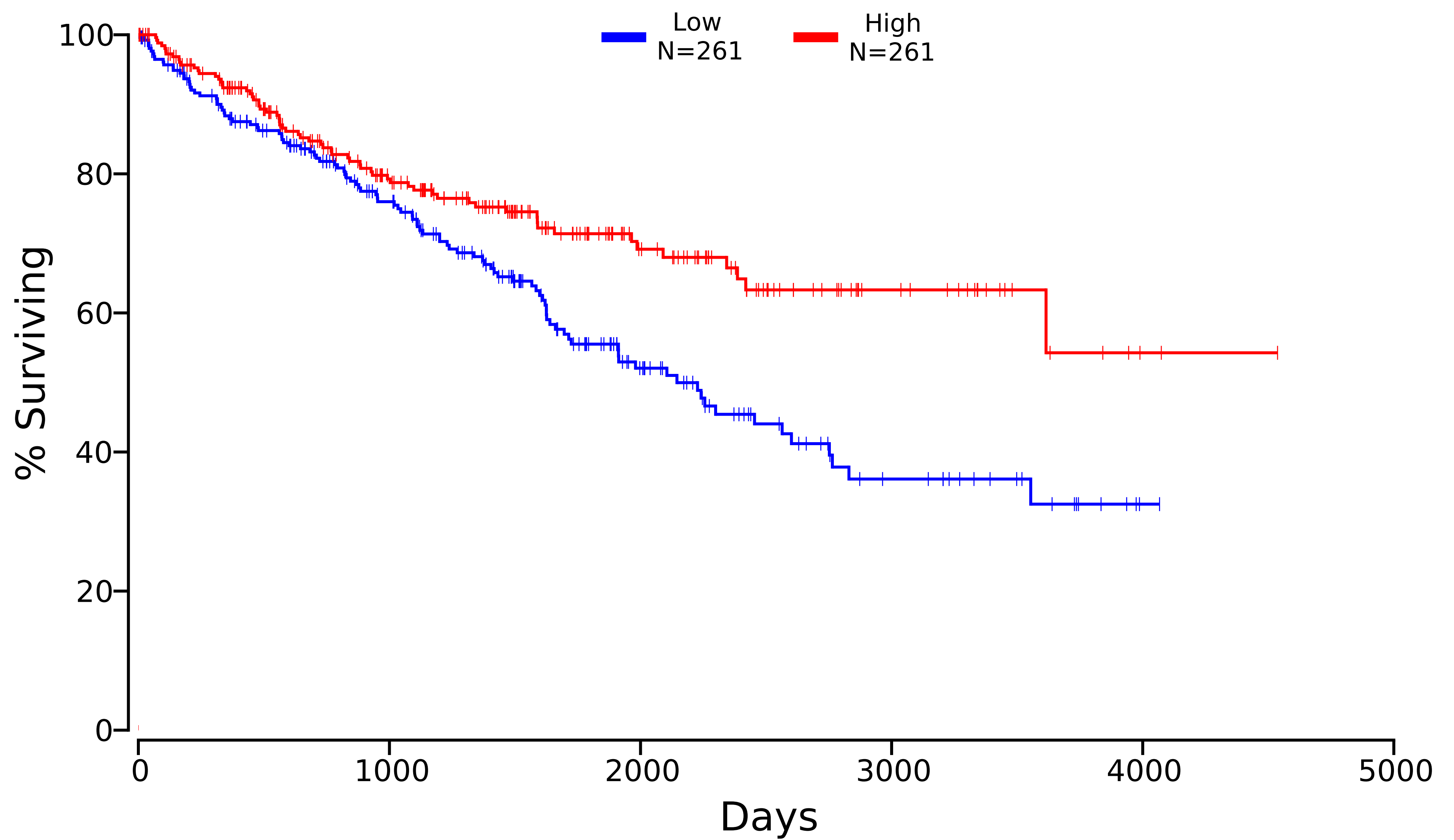

Supplement: Supplementary file 2 — Supplementary Information 2. [file 41598_2020_71997_MOESM2_ESM.zip › Suppl figure KM plot/CDH16_KIRC_1014_50_50.pdf]
